# The Use of the Aspirator in Retention of Urine

**Published:** 1886-12

**Authors:** W. Fairbanks

**Affiliations:** Wells


### THE USE OF THE ASPIRATOR IN RETENTION OF URINE. By W. FAIRBANKS, M.D. Edin., Wells.

In connection with the use of the aspirator, the following case presents some features of interest which seem to make it worth recording.

A. B., æt. 76, after suffering during several weeks from slight troubles of micturition, was on February 17th, 1886, seized with complete retention, and was relieved by catheter. Similar relief was given on several occasions, but three days later difficulty arose in passing an instrument, and the assistance of a hospital surgeon was obtained to overcome it.

On the 22nd February, symptoms of bladder distension were urgent, and now the difficulty of reaching the urine by the urethra was insuperable. The aspirator was used at 9 p.m., February 22nd, for the first time. As I am recording the case simply for its bearing on the use of the aspirator, I shall not enter into it fully, but simply enumerate the operations which were performed after the case came under my own observation.

| | | | | | | |
|---|---|---|---|---|---|---|
| Feb. 22. | 9.0 p.m. | Aspiration | | Mar. 1. | 9.30 a.m. | Aspiration |
| 23. | 4.0 p.m. | " | | | 8.0 p.m. | " |
| | 8.30 p.m. | " | | 2. | 9.0 a.m. | " |
| 24. | 1.0 a.m. | " | | | 8.15 p.m. | " |
| | 8.30 a.m. | " | | 3. | 9.45 a.m. | " |
| | 2.30 p.m. | " | | | 8.30 p.m. | " |
| | 8.0 p.m. | " | | 4. | 10.30 a.m. | Catheterism |
| 25. | 3.0 a.m. | " | | | 11.50 p.m. | Aspiration |
| | 10.0 a.m. | " | | 5. | 12.15 p.m. | " |
| | 5.0 p.m. | " | | 6. | 12.15 a.m. | " |
| | 11.50 p.m. | " | | | 11.15 a.m. | " |
| 26. | 10.30 a.m. | " | | | 9.30 p.m. | " |
| | 8.30 p.m. | " | | 7. | 1.0 p.m. | " |
| 27. | 9.30 a.m. | " | | 8. | 1.0 a.m. | " |
| | 7.30 p.m. | " | | | 9.30 a.m. | " |
| 28. | 12.0 noon | " | | 11. | 11.30 a.m. | " |
| | 8.30 p.m. | " | | | | |

It will be seen that during fifteen consecutive days, the whole of the urine, with the exception of that drawn off on one occasion by catheter, was passed through the needle of the aspirator. Upon some occasions, when the patient was more fully conscious, the skin was frozen before the puncture was made; but the pain caused by the needle was never greatly complained of, while the annoyance of the spray was not altogether trifling. From first to last the bladder was aspirated above the pubes thirty-two times. After the resumption of the catheterism on the 8th March, the aspirator was called for only once, and then by the want of prompt success with the catheter, and the dread of irritating the urethra just as it was becoming more tolerant of instrumentation.

The peculiar difficulties of the case were met by manœuvres familiar to most surgeons, and therefore needless to record; but it may be interesting to note two points: 1. It was probably due to the previous injection of a solution of cocaine that I was able to pass a No. 8 silver catheter on the 4th March. 2. A plan I found useful in a somewhat later stage of the case was as follows: A soft rubber catheter was passed down the urethra until it met with its check at or about the prostate. Then a long stylet, made for the purpose and shaped as desired, was passed within the catheter, which by its help was guided onwards into the bladder, with the minimum of irritation to the parts, and could be left in if desired.

Before passing on to consider the bearing of the case upon aspiration of the bladder, I may say that the patient gradually obtained control over his bladder, recovered, and is still (October) quite well. It is worth noting also, that when he had regained sufficient control to dispense with aid, and while the urine was ammoniacal and loaded with pus, it seemed, to all the surgeons who saw him, desirable to wash out the bladder from time to time. Nevertheless, as the proceeding was objectionable on many grounds, I first gave a trial to fluid extract of Corn silk; and while full doses of this drug were being given, the urine gradually cleared up, and in eight or nine days was free from deposit and from offensive smell.

The successful issue of this case would seem to indicate that the relief of the bladder by aspiration through the abdominal wall is at once—

*a.* Easy.
*b.* Perfectly safe.
*c.* Capable of very frequent repetition.

*a. Easy.*—Truly, nothing is easier than to puncture the over-distended bladder in the ample space provided by its very distension. But in a case of this kind there comes a time when the viscus is less tolerant of its contents, the urine is alkaline and loaded with pus, and the patient, with only six or seven ounces in his bladder, calls loudly for relief. If, under such conditions, one should attempt to aspirate, it is quite possible that no urine would flow. The flaccid bladder is not so easily pierced, and may escape puncture. A bold and quick thrust in the right direction, however, will not fail; and immense relief follows the removal of six or seven ounces of purulent and bloody urine, and the washing out the bladder through the aspirator with Sir Henry Thompson's solution. It is granted that cases presenting such a combination of circumstances as to require this proceeding must be very rare.

*b. Perfectly safe.*—Thirty-two aspirations performed, with nothing but benefit accruing, suggest the harmlessness of the proceeding. And yet the evidence is anything but conclusive. Indeed, to my mind, a series of ten cases of three aspirations apiece would be more to the purpose. It is always possible that inflammation may arise at the seat of the puncture, as in a case recorded by Dr. Macfie Campbell (*B. M. J.*, February, 1880), though such inflammation would seldom lead to similar disastrous consequences. Dr. Campbell, attributing the abscess in his case to extravasation of urine into the tissues, deduces the lesson that after aspiration the bladder should be kept undistended. In the case now recorded, the bladder was extremely distended before the second aspiration, and fully so on several subsequent occasions. Nevertheless, no urine escaped; nor can I think extravasation more than barely possible, if a moderately fine needle has been used. I can more easily conceive other causes for abscess at the seat of puncture. Though we may not go so far as to claim perfect safety for the operation, it is yet the safest and most suitable in this and in similar cases. Against tapping by the rectum there are objections: it cannot be repeated frequently; it is extremely difficult to keep a cannula in position, and very wretched for the patient if achieved. The elastic catheter of Mr. Davey (*B. M. J.*, December, 1875), though theoretically admirable, could not have been retained an hour in this case, owing to the restless and mischievous delirium of the patient.

*c. Capable of frequent repetition.*—Apart from the local skin-irritation produced, there seemed to be no limitation to the bladder's tolerance of aspiration. I find it stated that the aspirator may be used three or four times successively if a case demands it; but it seems probable that, if three or four aspirations are well borne, twelve or twenty may be practised without fear. No preconceived rules as to the frequency for its need should influence us. We should operate when the circumstances require it. Healthy kidneys may secrete with enormous rapidity after an over-distended bladder has been relieved for the first time; but this rush soon subsides, and then twelve hours will not serve to distend the bladder so fully as four hours would do at first. Unless the patient dread the prick of the needle, it is doubtless better to avoid freezing the skin in cases likely to require frequent operation.

It may be concluded that the surgeon may undertake aspiration of a distended bladder above the pubes with as much freedom from anxiety as attends any of the minor operations. He may thus gain time to conceive and carry out any plan for the permanent relief of his patient; and he need never become flurried or desperate over the tightest stricture, the most obstinate spasm, or the least accommodating prostate.

